# Effects of Antioxidants in Fermented Beverages in Tissue Transcriptomics: Effect of Beer Intake on Myocardial Tissue after Oxidative Injury

**DOI:** 10.3390/antiox12051096

**Published:** 2023-05-13

**Authors:** Alex Gallinat, Gemma Vilahur, Teresa Padro, Lina Badimon

**Affiliations:** 1 Cardiovascular Program-ICCC, IR-Hospital Santa Creu i Sant Pau, IIB-Sant Pau, IIBSANTPAU, 08041 Barcelona, Spain; 2 Centro de Investigación Biomédica en Red Cardiovascular (CIBER-CV), Instituto de Salud Carlos III, 28029 Madrid, Spain; 3 Cardiovascular Research Chair, Universitat Autònoma de Barcelona (UAB), 08193 Barcelona, Spain

**Keywords:** myocardial infarction, hypercholesterolemia, beer, alcohol, polyphenols

## Abstract

Fermented beverages, such as wine and beer, are rich in polyphenols that have been shown to have protective effects against oxidative stress. Oxidative stress plays a central role in the pathogenesis and progression of cardiovascular disease. However, the potential benefits of fermented beverages on cardiovascular health need to be fully investigated at a molecular level. In this study, we aimed at analyzing the effects of beer consumption in modulating the transcriptomic response of the heart to an oxidative stress challenge induced by myocardial ischemia (MI) in the presence of hypercholesterolemia in a pre-clinical swine model. Previous studies have shown that the same intervention induces organ protective benefits. We report a dose-dependent up-regulation of electron transport chain members and the down-regulation of spliceosome-associated genes linked to beer consumption. Additionally, low-dose beer consumption resulted in a down-regulation of genes associated with the immune response, that was not shown for moderate-dose beer consumption. These findings, observed in animals having demonstrated beneficial effects at the organ-level, indicate that the antioxidants in beer differentially affect the myocardial transcriptome in a dose-dependent manner.

## 1. Introduction

Fermented beverages, due to their plant-based origin, are rich in polyphenols and other bioactive compounds that have been linked to beneficial effects on cardiovascular health [1,2]. On the contrary, spirits and liquors are the product of a distillation process under high temperatures, causing a partial or total thermal degradation of their phenolic components and other bioactive compounds. Meta-analyses have shown a J-shaped association between cardiovascular risk and alcohol consumption for fermented beverages, such as wine and beer, but not for spirits [3,4,5]. Thus, while a low-to-moderate consumption of fermented beverages may associate with reduced cardiovascular risk, higher consumption levels would abrogate protection or even increase the risk of cardiovascular disease (CVD). The polyphenolic component of fermented beverages could explain the beneficial effects on cardiovascular health [6,7,8].

Polyphenols are a large and diverse group of natural compounds found in plants that are characterized by the presence of multiple phenol groups in their molecular structure and display several protective effects against oxidative stress [9]. Polyphenols can directly scavenge reactive oxygen species (ROS), chelate metal ions –involved in the generation of ROS–, and modulate cell signaling pathways participating in the antioxidant defense. Additionally, some polyphenols have been shown to induce the expression of antioxidant enzymes such as superoxide dismutase, catalase, and glutathione peroxidase [10]. Despite their potential to prevent or mitigate oxidative stress and associated diseases, the in vivo effects of polyphenols may be complex and dependent on many factors, such as dietary dose, bioavailability, type, and an individual’s genetic background and lifestyle [11]. Therefore, the potential effects of polyphenol-rich nutritional interventions should be assessed in vivo in clinically relevant models of oxidative stress-related disease.

Oxidative stress, defined as the unbalance between ROS production and the endogenous antioxidant defense, is a unifying mechanism for many cardiovascular disease risk factors [12]. It also significantly contributes to the pathogenesis and progression of CVDs, including myocardial ischemia (MI) and post-MI heart remodeling. Hypercholesterolemia (HC), which affects around 54.5% of U.S. adults, is a condition characterized by high levels of cholesterol in the blood [13]. It is a major risk factor for developing atherosclerosis and, therefore, MI [14]. HC is associated with higher oxidative stress, and consequently, its effects on cardiovascular health and disease span beyond the increased risk of MI [15,16]. In such a way, high cholesterol levels have been associated with larger infarcts resulting from an exacerbated inflammatory response [17], enhanced myocardial apoptosis [18], and an increased risk of thrombotic-related no-reflow phenomenon during reperfusion [19]. Clinical trials have also suggested that HC may increase the risk of heart failure and mortality in patients after MI [13].

The identification of the molecular components behind the protective properties of beer, and the deciphering of the mechanisms at play may bring the possibility to design new pharmacological and nutritional interventions to improve cardiovascular risk and post-MI outcomes.

In this study, we aimed to analyze the effects of beer consumption in modulating the heart’s transcriptomic response to an oxidative stress challenge induced by MI in a pre-clinical swine model. In previous studies, the same intervention design demonstrated physiological organ-level benefits, such as a global improvement of the myocardial performance, a reduction of the final myocardial damage size, and a reduction of the systemic oxidative stress induced by myocardial ischemia [20]. In this study, we hypothesized that beer intake might convey modifications in the myocardial transcriptome that would explain the cardiac organ effects via an improved antioxidant defense. HC was included as a common co-morbidity of MI, and two different beer consumption regimes were considered to evaluate potential dose-dependent effects.

## 2. Materials and Methods

### 2.1. Experimental Design

Experimental procedures were reviewed and approved by the Institutional Animal Care and Use Committees (CEEA-IR) and authorized by the Animal Experimental Committee of the local government (#9340) in accordance with the Spanish law (RD 53/2013) and European Directive 2010/63/EU. In addition, the investigation conforms to the Guide for the Care and Use of Laboratory Animals published by the U.S. National Institutes of Health (NIH Publication No. 85-23, revised 1985) and follows the ARRIVE 2.0 guidelines.

Commercial cross-bred four-month-old female pigs (*n* = 23) were fed a western-type hypercholesterolemic diet (20% saturated fat, 2% cholesterol, 1% cholic acid) and randomized to the following groups: (I) Low-dose beer group, which received 12.5 g alcohol/day (LDB, *n* = 7). (II) Moderate-dose beer group, which received 25 g alcohol/day (MDB, *n* = 7). (III) Control group, which only received a hypercholesterolemic diet with no beer (Control, *n* = 9). Ten days after feeding, animals were subjected to a close-chest mid-left anterior descending coronary artery balloon occlusion for 90 min as previously described [20] and sacrificed 21 days after MI. Diet specifications were maintained at all times. Daily beer doses were distributed in two portions, one given in the morning and the other one in the afternoon, both coinciding with the feeding time. At the beginning of the study, animal weights were similar across groups: control, 35.1 ± 1.0 kg; LDB, 34.0 ± 1.3 kg; MDB, 35.6 ± 1.3 kg.

### 2.2. Animal Model of MI

MI was induced as previously described, by close-chest mid-left anterior descending coronary artery total balloon occlusion for 90 min [20]. Briefly, animals received a loading dose of clopidogrel 12 h prior to the procedure. Anesthesia was induced with a mixture of tiletamine, zolazepam (7 mg/kg), and medetomidine (0.07 mg/kg). Animals then underwent endotracheal intubation, and anesthesia was maintained with isofluorane inhalation (2%). A continuous infusion of amidorane (300 mg, 75 mg/h) and lidocaine (150 mg, 37.5 mg/h) were administrated as prophylaxis for malignant ventricular arrhythmias. The whole procedure was performed under electrocardiographic and hemodynamic monitoring, and successful total occlusion of the mid-left coronary artery was confirmed by contrast injection. Animals were then allowed to recover and kept under the initial diet specifications for 21 days.

### 2.3. Sample Collection

At sacrifice, 21 days after MI, animals were anesthetized, and a balloon catheter was placed and inflated within the same place as for the MI induction. Evan’s blue dye was then injected into the coronary sinus to outline the myocardium at risk. Immediately after the injection, hearts were arrested, with an intravenous administration of 10 mL of KCl 2M, excised, and cross-sectioned. Samples were then taken from the myocardium at risk (i.e., viable post-ischemic tissue), snap-frozen in liquid nitrogen, and stored at −80 °C until processing for the following analyses.

### 2.4. Sample Processing

Frozen myocardial tissue samples were ground to a powder using a mortar and pestle, and total RNA was extracted using the RNesasy Microarray Tissue Mini Kit (Qiagen; Valencia, CA, USA). RNA quantification was determined spectrophotometrically with a Nanodrop ND-1000 system (Thermo Fisher Scientific, Waltham, MA, USA). RNA quality was measured using the Agilent 2100 Bioanalyzer technology (Agilent Technologies; Santa Clara, CA, USA) with the Agilent RNA 6000 Nano Kit (Agilent Technologies; Santa Clara, CA, USA). Only RNA samples with RIN > 7 values (RNA integrity number) were chosen for transcriptomic analysis. All processed samples were stored at −80 °C until used.

### 2.5. Transcriptomics and In Silico Analysis

Using the GeneChip 3′ IVT Express kit (Affymetrix, Santa Clara, CA, USA) 200 ng of total RNA (mixed with poly-A controls; Affymetrix, Santa Clara, CA, USA) were converted to double-strand DNA, in two steps, in order to obtain aRNA by an in vitro transcription during 4 h. 12 µg of purified aRNA were fragmented and labeled with biotin using the WT Terminal Labeling Kit (Affymetrix, Santa Clara, CA, USA). Hybridization controls were added, and samples were then processed with the Hybridization, Wash and Stain Kit (Affymetrix, Santa Clara, CA, USA) and hybridized to a GeneChip Porcine Genome Array (Affymetrix, Santa Clara, CA, USA) for 16 h at 45 °C and 60 rpm, according to manufacturer’s instructions. Washing, staining, and scanning of microarrays were performed according to Affymetrix’ instructions using the Affymetrix GeneChip 3000 7G System (Hybridization Oven 640, Fluidic Station 450 and GeneChip 3000 7G Scanner; Affymetrix, Santa Clara, CA, USA). Raw gene expression levels were then background corrected, normalized, and summarized following the robust multichip average (RMA) strategy. Because of the large number of unassigned probes in the official Affymetrix’ annotation file, a custom annotation file was generated combining the last official release, and the annotation file generated by Naraballobh et al. [21] (Supplementary Materials File S1). All data pre-processing and in silico analyses were performed using RStudio (RStudio, Boston, MA, USA).

### 2.6. Statistical Analysis

After data pre-processing, differential expression changes for each gene were calculated using the ”limma” R package [22]. Briefly, a linear model was fitted to the data, including the experimental design, and empirical Bayes moderation was used to estimate the variance and test for differential expression. The resulting p-values were adjusted for multiple comparisons following the Benjamini–Hochberg procedure, and false discovery rates (FDR) were considered for assessing significance. The final dataset was then functionally annotated for Gene Ontology biological processes terms following a gene-set enrichment analysis (GSEA) strategy [23], using the ”ClusterProfiler” R package [24]. For the core enrichment analyses and dose-dependent effects, normality was assessed with the Shapiro–Wilk method. When data were normally distributed, a two-tailed Student’s *t*-test was employed to assess statistical significance. Otherwise, a Mann-Whitney test was used. All statistical analyses were performed in RStudio (RStudio, Boston, MA, USA). 

## 3. Results

### 3.1. Beer Intake Modifies the Global Gene Expression Profile of the Myocardium

**Figure 1:**
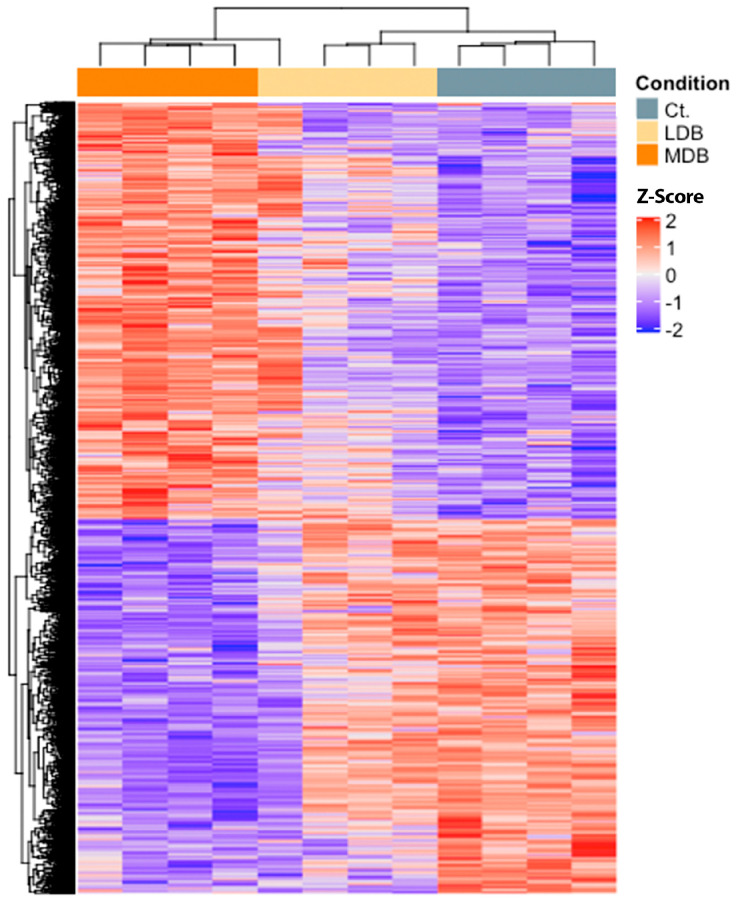
Effect of low and moderate-dose beer consumption regimes upon the myocardium transcriptome, 21 days post-myocardial infarction in hypercholesterolemic pigs. Heatmap representation of the top 100 most differentiated transcripts across groups. LDB, low-dose beer; MDB, moderate-dose beer; Ct., control.

Changes in the gene expression profile of the post-ischemic myocardium –myocardium at risk– were evaluated at 21 days post-MI following an expression array approach. As depicted in Figure 1, beer consumption modified the heart transcriptome in a dose-dependent manner. More specifically, while a low-dose beer regime (12.5 g alcohol/day) slightly affected the heart’s gene expression profile, a moderate-dose beer intake (25 g alcohol/day) resulted in a greatly differentiated transcriptome compared to the control (Figure 1). The top 10 differentially expressed genes for each group comparison are depicted in Supplementary Materials File S2.

### 3.2. Beer Consumption Up-Regulates Electron Transport Chain Members and Down-Regulates Spliceosome-Associated Genes

**Figure 2:**
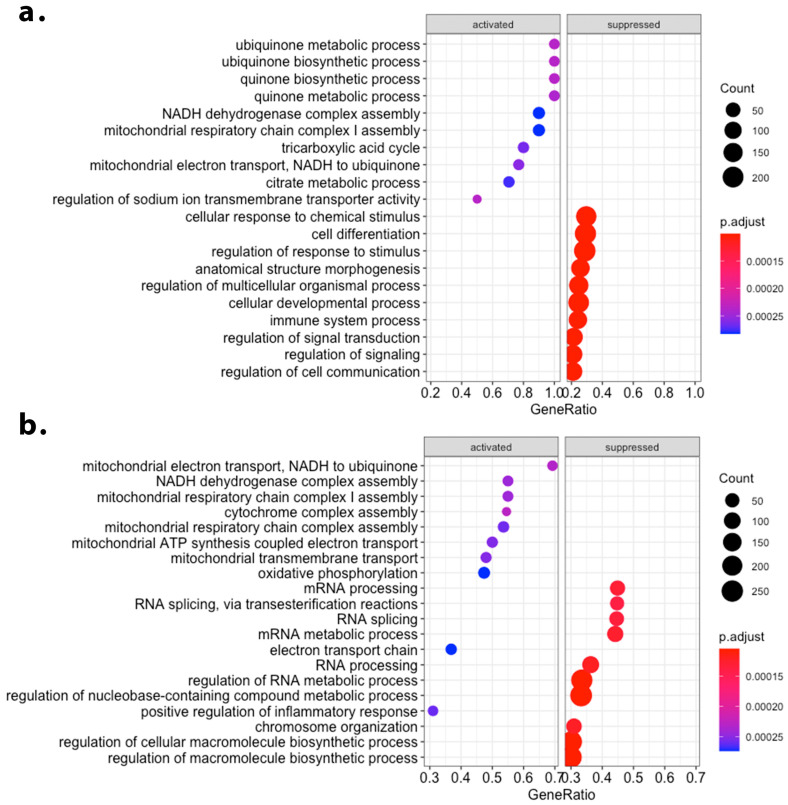
Myocardial transcriptomic effect of beer consumption in a hypercholesterolemic swine model of infarct healing, 21 days post-myocardial infarction. Gene-set enrichment analysis result for Gene Ontology biological process terms. The top 10 activated and suppressed terms detected to be significantly enriched in the dataset are depicted alongside the gene ratio for the enrichment, count, and adjusted *p*-value, for (**a**) a low-dose beer consumption regime, and (**b**) a moderate-dose beer consumption regime. ”GeneRatio” refers to the ratio between differentially expressed genes belonging to a biological process and the total number of genes listed within the same biological process term.

**Figure 3:**
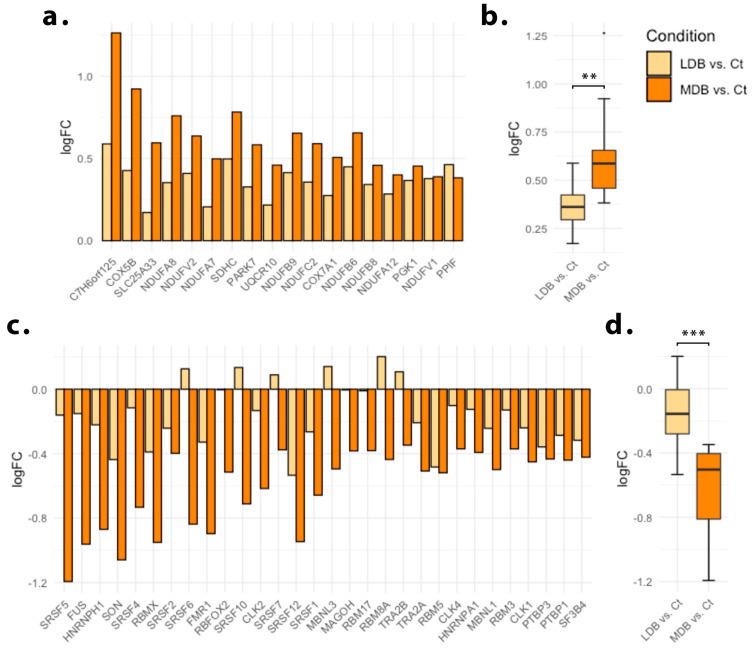
Leading edge analysis. Myocardial transcriptomic effect of beer consumption upon the genes contributing to ”oxidative phosphorylation” and ”RNA splicing” GeneOntology biological process terms enrichment result. (**a**) Log fold-change (LogFC) of genes contributing to the ”oxidative phosphorylation” term enrichment. (**b**) Pooled LogFC of all genes contributing to the ”oxidative phosphorylation” term enrichment. (**c**) LogFC of genes contributing to the ”RNA splicing” term enrichment. (**d**) Pooled LogFC of all genes contributing to the “RNA splicing” term enrichment. Myocardial gene expression was assessed 21 days after myocardial ischemia. All animals were fed a western-type hypercholesterolemic diet plus low-dose beer (LDB), moderate-dose beer (MDB), or no beer, (control, Ct). ** *p* < 0.01, *** *p* < 0.001.

We next explored Gene Ontology biological process term enrichments, following a GSEA approach and ranking genes by fold-change relative to the control group. As a result, the induction of genes involved in the electron transport chain was the most consistently detected enrichment for both low and moderate-dose beer regimes (Figure 2). Interestingly, the moderate-dose beer regime also resulted in the down-regulation of genes associated with the spliceosome and RNA processing (Figure 2b). In order to analyze putative dose effects upon these gene sets, we performed a leading-edge analysis for both ”oxidative phosphorylation” and ”RNA splicing” Gene Ontology terms. We identified a direct dose-dependent effect for both gene sets (Figure 3).

### 3.3. Low and Moderate-Dose Beer Regimes Have Opposite Effects upon Post-MI Immune Response

**Figure 4:**
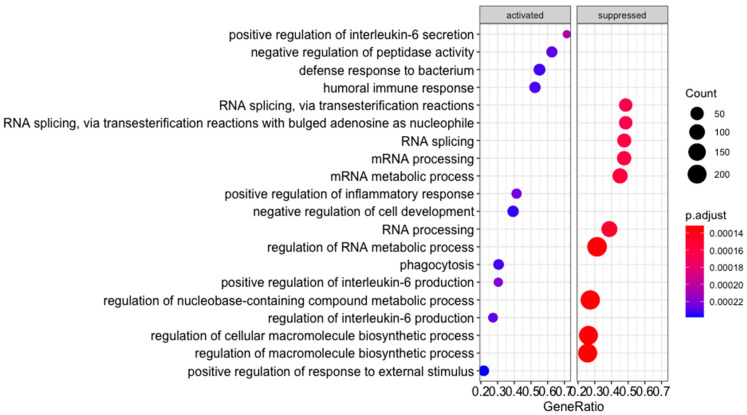
Myocardial transcriptomic effect of different beer consumption regimes in a hypercholesterolemic swine model of infarct healing, 21 days post-myocardial infarction. Gene-set enrichment analysis result for Gene Ontology biological process terms. The top 10 activated and suppressed terms detected to be significantly enriched in the dataset are depicted alongside the gene ratio for the enrichment, count, and adjusted *p*-value, for a moderate-dose beer consumption regime compared to a low-dose beer consumption regime. ”GeneRatio” refers to the ratio between differentially expressed genes belonging to a biological process and the total number of genes listed within the same biological process term.

**Figure 5:**
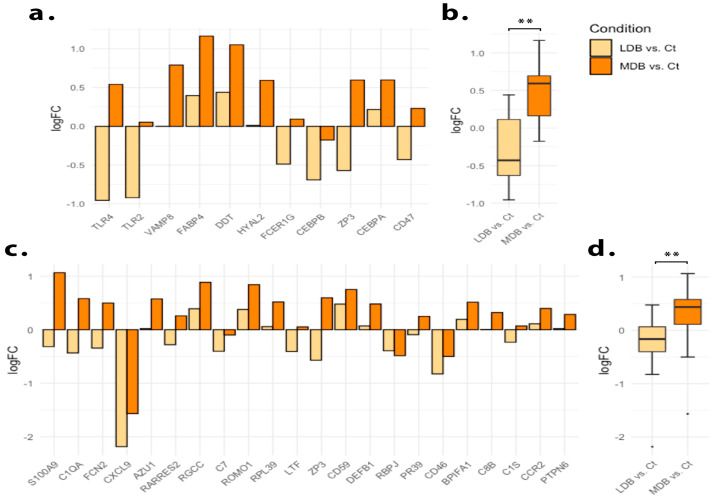
Leading edge analysis. Myocardial transcriptomic effect of beer consumption upon the genes contributing to ”positive regulation of inflammatory response” and ”humoral immune response” GeneOntology biological process terms enrichment result. (**a**) Log fold-change (LogFC) of genes contributing to the ”positive regulation of inflammatory response” term enrichment. (**b**) Pooled LogFC of all differentially expressed genes contributing to the ”positive regulation of inflammatory response” term enrichment. (**c**) LogFC of genes contributing to the ”humoral immune response” term enrichment. (**d**) Pooled LogFC of all differentially expressed genes contributing to the ”humoral immune response” term enrichment. Myocardial gene expression was assessed at 21 days after myocardial ischemia. All animals were fed a western-type hypercholesterolemic diet plus low-dose beer (LDB), moderate-dose beer (MDB), or no beer, (control, Ct). ** *p* < 0.01.

**Figure 6:**
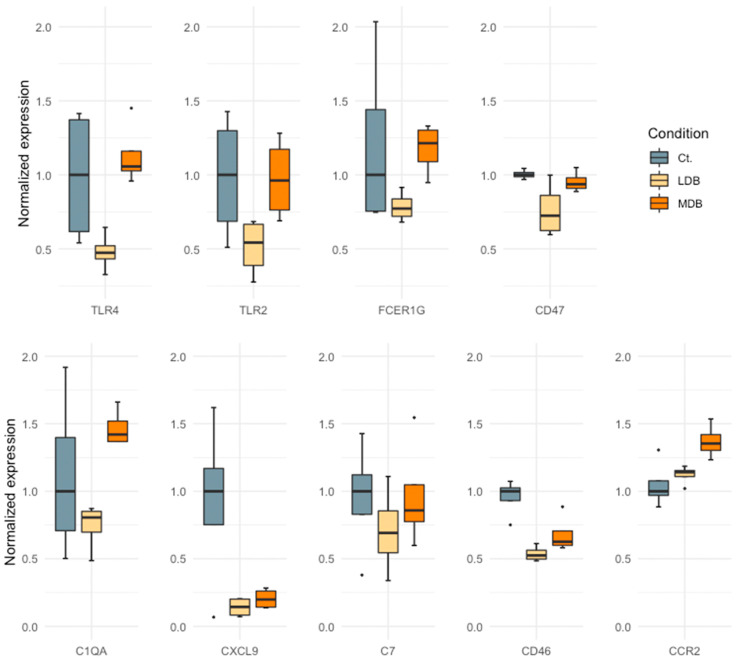
Myocardial transcriptomic effect of different beer consumption regimes in a hypercholesterolemic swine model of infarct healing, 21 days post-myocardial infarction. Gene expression of selected inflammatory markers was normalized to the control group expression value. TLR4, Toll-Like Receptor 4; TLR2, Toll-Like Receptor 2; FCER1G, High Affinity Immunoglobulin Epsilon Receptor Subunit Gamma; CD47, Integrin-Associated Protein; C1QA, Complement C1q A Chain; CXCL9, C-X-C Motif Chemokine Ligand 9; C7, Complement C7; CCR2, C-C Motif Chemokine Receptor 2. All animals were fed a western-type hypercholesterolemic diet plus low-dose beer (LDB), moderate-dose beer (MDB), or no beer, (control, Ct).

To analyze differences between beer doses upon the post-MI heart transcriptome, we ran another GSEA ranking genes by fold-change between moderate and low-dose beer regimes. As a result, positive enrichments of gene sets associated with the immune response were significantly detected (Figure 4). To evaluate the impact of different levels of beer consumption on the immune response, we conducted a leading-edge analysis for the Gene Ontology terms ”positive regulation of inflammatory response” and ”humoral immune response”, and found opposite effects for low and moderate-dose beer regimes (Figure 5). While a low-dose beer consumption regime had an overall inhibitory effect on genes associated with the immune response, a moderate dose stimulated their expression. Notably, a J-shaped association between beer consumption and the normalized expression of the selected immune response-related markers was found (Figure 6).

## 4. Discussion

In the present study, we aimed to investigate the effects of beer consumption in modulating the transcriptomic response of the cardiac tissue to an oxidative stress challenge induced by ischemia in the presence of HC in a pre-clinical swine model. Our approximation allowed us to screen for whole-beer effects on the heart’s response to MI regardless of the effects associated with the alcoholic fraction or the polyphenolic component of beer. Although the deciphering of the molecular basis of the reported effects is a scientifically relevant issue, especially for the identification of novel therapeutic interventions, the recognition and understanding of the broad molecular effects of beer is a matter of interest. Our results showed that beer consumption up-regulated electron transport chain members and down-regulated spliceosome-associated genes, both in a dose-dependent manner. Furthermore, opposite dose effects were found for low and moderate-dose beer regimes upon the expression of genes related to the immune response.

Inflammation and the immune response play a significant role in the healing and remodeling process following MI. During the early stages of MI, inflammation is activated in response to tissue injury and necrosis [25]. This process involves leukocyte infiltration and the release of pro-inflammatory cytokines and other mediators. The main goal of the initial inflammatory response is the removal of dead cells and matrix debris, as well as the activation of a reparative program [26]. However, an excessive or prolonged inflammatory response can cause additional damage and impair the healing process, playing a role in the pathogenesis of heart failure [27,28]. Importantly, both pro- and anti-inflammatory signals are required for the correct resolution of myocardial injury [29,30]. At 21 days after MI, the healing process is typically in the later stages of tissue repair and remodeling. Failure of an appropriate resolution of the inflammatory response post-MI is associated with unsuccessful left ventricular remodeling and underlies heart failure [31]. Elevated levels of inflammatory markers have been associated with poor prognosis in the development of heart failure and a greater risk of future coronary events [27]. Therefore, a lower inflammatory state of the heart in the later stages of infarct healing may associate with better outcomes. A low-dose beer consumption regime resulted in the down-regulation of inflammatory markers in the myocardium 21 days post-MI, whereas the effect was not evident at a moderate-dose beer consumption regime. These results coincide with the widely acknowledged J-shaped association between cardiovascular risk and the consumption of fermented beverages, where low-to-moderate consumption regimes associate with reduced cardiovascular risk, and protection is lost at higher consumption levels [3,4,5]. However, the mechanism by which different doses result in opposite effects is still unknown. Nevertheless, the reported inhibitory effects of low-dose beer regime on inflammation and the immune response are likely to arise from the polyphenolic component of beer, as previous research has shown the inhibition of the inflammasome pathway for low-to-moderate beer consumption regimes, for both alcohol-free and traditional beer [32].

We also report a dose-dependent up-regulation of electron transport chain members, especially for genes associated with the mitochondrial complex I. These effects may arise from the polyphenolic component of beer, ethanol, or a combination of both. On the one hand, the metabolism of small amounts of ethanol may result in an increased generation of NADH which in turn could lead to metabolic adaptations of the mitochondria. Although the liver is the major alcohol-metabolizing organ, ethanol easily distributes throughout the body reaching virtually all tissues, including the heart [33]. Ethanol metabolism begins with the oxidation of ethanol to acetaldehyde throughout the alcohol dehydrogenase, which generates cytosolic NADH. Later on, acetaldehyde is metabolized to acetate through the mitochondrial low-KM aldehyde dehydrogenase, generating further NADH. The resulting acetate will eventually reach the tricarboxylic acid cycle, after its conversion to acetyl-CoA [34]. An increased generation of NADH associated with alcohol consumption entails a metabolic challenge for the mitochondria and an increased production of ROS, through electron leakage at the mitochondrial complex I [35]. While the accumulation of ROS has a number of detrimental effects on macromolecule structure and function, ROS in small amounts has been identified to play a pivotal role in the establishment of cardioprotection in ischemic conditioning studies [36,37]. When below a threshold for cellular stress, ROS may trigger an array of bioenergetics adaptations in the mitochondria resulting in increased ATP production and overall cell protection [38]. Importantly, while the brain and liver have been reported to decrease both the expression of electron transport chain members and the mitochondrial content in response to chronic ethanol exposure [39,40], heart mitochondria respond differently, stimulating mitochondrial biogenesis [41]. Given that mitochondria are central players of MI-induced damage, the reported up-regulation of mitochondrial complexes may contribute to the previously reported improvement of cardiac performance post-MI for the beer-fed groups [20]. 

On the other hand, previous research has shown diverse mitochondrial effects of polyphenols which may also explain the reported effects. To this extent, polyphenols have been recognized to modulate mitochondrial biogenesis, mitochondrial membrane potential, electron transport chain activity, redox status, and mitochondria-triggered apoptosis [42,43,44]. Yet, polyphenols are a large and diverse group of bioactive compounds, and therefore, it can be challenging to determine their specific effects. 

The reported down-regulation of spliceosome-associated genes is of particular interest, as splicing is a key process in the regulation of gene expression and has been shown to play a role in the development of heart failure [45,46]. To the best of our knowledge, this is the first study to report the effect of beer consumption on the splicing machinery of the heart. Importantly, RNA splicing is a complex and tightly regulated process that involves a close interaction between genetic and epigenetic machinery. Therefore, it would be presumptuous to advance whether this effect would have a positive or negative impact on heart performance and cardiovascular risk before an appropriate investigation. The potential implications of these results on the MI healing process and the development of heart failure deserve further investigation.

The present work follows a previous study where the same intervention design demonstrated physiological organ-level benefits together with an increased antioxidant capacity of the high density lipoproteins particles (HDLs) and decreased oxidative stress 21 days post-MI [20]. An alcohol-free beer group was included and also associated with anti-oxidation. Thereby, these effects are likely to arise from the polyphenolic fraction of beer. Importantly, oxidative stress is a unifying mechanism for many cardiovascular disease risk factors [12], and a widely acknowledged player in the pathogenesis and progression of CVDs. Therefore, the results here reported may arise from the effects of polyphenols on the systemic oxidative stress induced by MI. Further research is needed in order to appropriately address the molecular basis of the reported effects.

Altogether, our results depict the effects of beer consumption modulating the heart’s response to an oxidative stress challenge induced by MI in the presence of HC. 

## 5. Conclusions

Overall, our findings provide insights into the potential mechanisms through which beer intake may affect the response of the heart to oxidative damage. We report that beer consumption had dose-dependent effects on the expression of genes related to the electron transport chain and the spliceosome, and dose-specific effects on inflammation and the immune response. Therefore, the data here presented supports the association between fermented beverage consumption in low-to-moderated amounts and beneficial cardiovascular effects. Deciphering the operating mechanisms behind the cardioprotective effects of low-to-moderate beer consumption may bring new therapeutic opportunities and the possibility of designing nutritional interventions to improve post-MI outcomes.

## Data Availability

All of the data is contained within the article and the Supplementary Materials.

## Supplementary Materials

The following supporting information can be downloaded at: https://www.mdpi.com/article/10.3390/antiox12051096/s1, File S1: Custom annotation file for the Affymetrix’ GeneChip Porcine Genome Array probes. File S2: Top 10 differentially expressed genes across all comparisons between control, low-dose beer, and moderate-dose beer groups.
